# The Arabidopsis Accessions Selection Is Crucial: Insight from Photosynthetic Studies

**DOI:** 10.3390/ijms22189866

**Published:** 2021-09-13

**Authors:** Joanna Wójtowicz, Katarzyna B. Gieczewska

**Affiliations:** Department of Plant Anatomy and Cytology, Faculty of Biology, University of Warsaw, I. Miecznikowa 1, 02-096 Warsaw, Poland; j.wojtowicz@biol.uw.edu.pl

**Keywords:** *Arabidopsis thaliana* accessions, Col-0, Col-1, Col-2, Col-8, Ler-0, Ws-2, Chlorophyll *a* fluorescence, photosynthetic efficiency

## Abstract

Natural genetic variation in photosynthesis is strictly associated with the remarkable adaptive plasticity observed amongst *Arabidopsis thaliana* accessions derived from environmentally distinct regions. Exploration of the characteristic features of the photosynthetic machinery could reveal the regulatory mechanisms underlying those traits. In this study, we performed a detailed characterisation and comparison of photosynthesis performance and spectral properties of the photosynthetic apparatus in the following selected *Arabidopsis thaliana* accessions commonly used in laboratories as background lines: Col-0, Col-1, Col-2, Col-8, Ler-0, and Ws-2. The main focus was to distinguish the characteristic disparities for every accession in photosynthetic efficiency that could be accountable for their remarkable plasticity to adapt. The biophysical and biochemical analysis of the thylakoid membranes in control conditions revealed differences in lipid-to-protein contribution, Chlorophyll-to-Carotenoid ratio (Chl/Car), and xanthophyll cycle pigment distribution among accessions. We presented that such changes led to disparities in the arrangement of the Chlorophyll-Protein complexes, the PSI/PSII ratio, and the lateral mobility of the thylakoid membrane, with the most significant aberrations detected in the Ler-0 and Ws-2 accessions. We concluded that selecting an accession suitable for specific research on the photosynthetic process is essential for optimising the experiment.

## 1. Introduction

The inbreeding annual plant *Arabidopsis thaliana* inhabits a wide range of climates across its native Eurasian range [1,2]. Arabidopsis specimens collected in nature that represent homozygous genotypes are called ecotypes. Nowadays, Arabidopsis ecotypes are known for their remarkable adaptability and a vast diversity of forms. The first Arabidopsis accessions subjected to experiments were collected near Limburg in Germany by a botanist Friedrich Laibach at the beginning of the 20th century [3]. The extensive seed collection (ecotypes and X-ray induced mutants) established by Professor Laibach served as the basis for the Arabidopsis Information Service (AIS) seed bank and eventually, with the support and interest of other scientists, for modern stock centres such as the NASC (Nottingham Arabidopsis Stock Centre, in 1990) and the ABRC (Arabidopsis Biological Resource Center, Columbus, in 1991) years later [4]. When the *Arabidopsis thaliana* genome was sequenced and published in 2000 using the Columbia accession, it triggered a wide range of genetic resources. Knockout mutants, transgenic overexpression lines, and recombinant inbred mapping populations have been developed and used to elucidate the architecture of developmental and physiological pathways underlying ecologically essential traits [5].

By now, there are almost 7000 Arabidopsis accessions available in stock centres with Columbia (Col-0 to Col-8), Wassilewskija (Ws-0 to Ws-4), and *Landsberg erecta* (Ler-0, Ler-1) being the most popularly used background lines in laboratories all over the world (NASC, arabidopsis.info, [6]). The Col and Ler-0 lines descend from Laibachs’ natural Landsberg strain (La), while the Ws line was initially collected in Wassilewskija, Russia. The Col-1 ecotype is considered the original nonirradiated line propagated by György Rédei and the source of subsequent Col-n lines, whereas Ler-0 was received via an X-ray mutagenesis and was introduced by a plant biologist, Willem Feenstra [5]. Nowadays, the Col, Ler-0, and Ws-2 serve as background lines for Salk T-DNA stocks, JIC Gene trap Ds lines, and Fieldman T-DNA tagged lines, respectively [7,8]. Although Arabidopsis mutants in plant biology are a valuable tool, they cannot be applied to define some aspects, e.g., regulatory mechanisms or signal transduction pathways. Thus, the analysis of the natural variation, adaptive plasticity, and quantitative genetics of *A. thaliana* accessions could explain these issues.

The chloroplast thylakoid membranes, where the photosynthetic machinery is assembled, create a well-organised environment divided into stacked membranes-grana and unstacked stroma thylakoids densely packed in proteins and lipids [9]. Chlorophyll-protein complexes (CP)- the main components are spatially separated into appressed grana stacks with Photosystem II (PSII), and light-harvesting complexes (LHCII) organised in LHCII–PSII supercomplexes and Photosystem I (PSI) and its antenna (LHCI) organised in LHCI–PSI supercomplexes present in stromal lamellae and grana end membranes [10]. The stabilisation of the photosynthetic apparatus is achieved via proper protein–lipid and protein–protein interactions, involving specific protein ordering and pigment location [11]. Carotenoids, essential constituents of the thylakoids, are primarily attached to core and antenna proteins of CP- complexes with a small, unbound pool present in the lipid matrix of the thylakoid membranes [12]. High protein densities and pigment content in thylakoids are favourable to efficient light absorption and harvesting to provide adequate energy transfer and boost photosynthetic efficiency [13]. Moreover, the cooperation between photosynthetic proteins, chlorophylls, and pigments maintains high thylakoid membrane flexibility and the ability to adapt to dynamically changing environmental conditions [14,15]. 

The light-dependent phase of photosynthesis is not flawlessly in terms of energy efficiency [16,17]. The vast majority of the available energy is re-emitted as heat or fluorescence due to specific bottlenecks in the very ability of the photosynthetic apparatus to absorb energy [18,19]. Improving the photosynthesis process itself and minimizing energy losses is essential for global food production in the currently changing climate [16]. A high temperature, often accompanied by high-light stress, quickly damages the photosynthetic apparatus [20,21]. High temperature causes disturbances in the fluid structure of the thylakoid membranes, leading, among others, to inhibiting the proper transport of electron carriers between the components of photosystems [22]. A short period of elevated temperature inhibits the synthesis of chlorophyll (Chl) in chloroplasts [23]. The sustained high temperature additionally promotes the process of Chl degradation and even ultimately impairs the Chl biosynthesis [23]. Damage to the water oxidizing complex PSII also occurs, contributing to the loss of structure and function of the entire machinery. Thus, the photoinhibition process begins to be observed [18,24,25]. Additionally, the accompanying burst of PSI production of reactive oxygen species damages proteins, membrane lipids, and thylakoids [26]. Attempts are made to increase the efficiency and stability of the photosynthesis process in plants and algae. Thanks to genetic engineering methods, it is possible to silence or overexpress genes of individual components of the photosynthetic system [16], such as increasing the number or size of external antennas that absorb light photons [17]. It does not always have to be a direct change, for example, the manipulation of CAO (chlorophyllide *a* oxygenase) activity—an enzyme involved in the synthesis of chlorophyll *b* leads to modification of the size and number of LHCII antennas as Chl*b* is their main component [17,27].

The adaptive plasticity of the photosynthetic machinery to various environmental conditions depends on the level of genetic variation for photosynthesis in a population [28]. Investigating the specific traits responsible for the natural genetic variation in photosynthesis will reveal the link between regulatory mechanisms and the interactions of photosynthetic phenotypes with the environment. *Arabidopsis thaliana* accessions with their high adaptive potential seem to be suitable for studying this topic. Numerous papers concerning changes in the compensation mechanism of the photosynthetic parameters in terms of adaptation amongst Arabidopsis accessions were investigated [6,29,30] with a two-way approach: studies based on available photosynthetic mutants in comparison with accession to reveal adaptation changes in the genome [31,32,33,34,35,36,37] and a parallel between accessions exposed to stressful abiotic conditions [38,39,40,41,42,43].

This study examined and compared the photosynthetic parameters of six selected, widely used Arabidopsis accessions: Col-0, Col-1, Col-2, Col-8, Ws-2, and Ler-0, which initially come from environmentally distinct regions. We report significant differences amongst accessions in the thylakoid lipid-to-protein proportion, chlorophyll, and carotenoid content, with the most prominent differences detected in Ler-0 and Ws-2. However, divergent values obtained for the Chl/Car, Lut/β–Car ratio, and protein-to-protein interactions led to disproportions in the CP complexes’ distribution and changes in membrane mobility with no harm to the overall photosynthetic efficiency. The control growth conditions chosen for this study aimed to closely elucidate even the most minor disparities in the photosynthetic apparatus composition between accessions, which could be beneficial for selecting an appropriate background line in mutant characterisation experiments and future adaptive traits investigations. 

## 2. Results

The main objective of this work was to elucidate the changes in the photosynthetic apparatus between six Arabidopsis ecotypes: Col-0 (N1092), Col-1(N3176), Col-2 (N907), Col-8 (N60000), Ws-2 (N22659), and Ler-0 (NW20). Col-1 is the original, homogenous Columbia ecotype line selected by György Rédei [5]. Therefore, in our study, the Col-1 line was chosen as a reference plant amongst picked accessions. 

### 2.1. Plant Phenotype, Efficiency of the Photosynthetic Apparatus, and the Chlorophyll–Protein Complexes’ Composition

**Figure 1:**
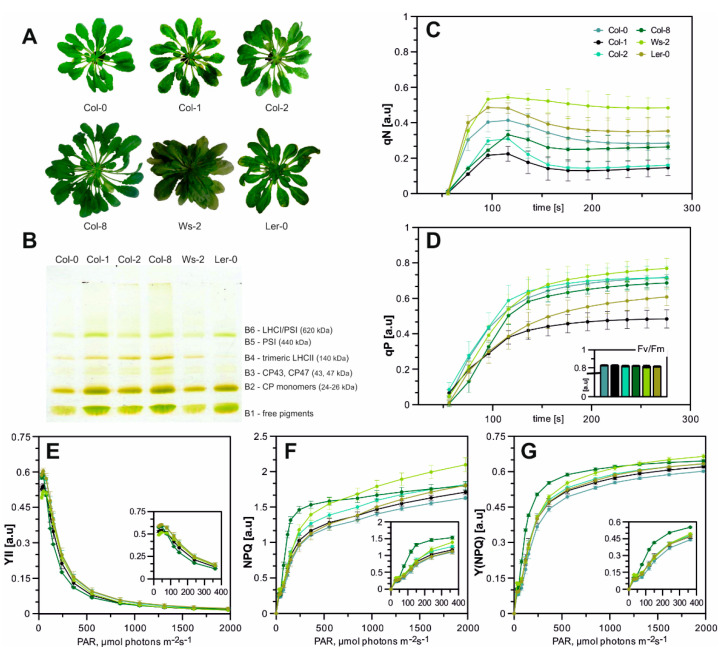
(**A**)—*A. thaliana* accessions after 8–10 weeks of growth in hydroponic culture (plants were grown for 8–10 weeks in an 8 h photoperiod at 22 °C/18 °C (day/night) at PAR 110 µmol photons m^–2^ s^–1^). (**B**)—Native-PAGE separation of CP complexes from thylakoid complexes. The picture shows true colours; the numbering of green bands (B1-B6) is reported; 8.3 µg of total chlorophyll was loaded into each well. The presented results are representative of at least five independent experiments. (**C**)—Stress-induced limitation of NPQ (qN) and (**D**)—photochemical fluorescence quenching (qP) of *A. thaliana* accessions; efficiency of PSII–Fv/Fm-insert bar chart, numerical values and SDs in Table A1. Error bars indicate standard deviations, n per measurement = 10. (**E**–**G**) Changes in photosynthetic parameters values. Small schemes indicate close-ups of the changes in the 0–400 PAR (µmol photons m^−2^ s^−1^) range. (**E**)—Effective quantum yield of PSII, Y(II). (**F**)—Non-photochemical quenching (NPQ). (**G**)—Quantum yield of regulated energy dissipation in PSII, Y(NPQ) measured under increasing light intensities. Data are mean values ± SD from at least three independent experiments.

All *A. thaliana* accessions have grown relatively evenly, although we noticed minor alterations in their morphology. Col-0, Col-1, and Col-8 had a long, thin proximal part of the leaf blade (Figure 1A), while Wasilevskija (Ws-2) was the one with a more densely packed rosette. *Landsberg erecta* (Ler-0) was more profound in the shade and slightly decoloured in normal light conditions. To describe the functional characteristics of leaves in all accessions, the main parameters of chlorophyll *a* (Chl*a*) fluorescence in vivo were measured. Obtained data revealed that the efficiency of PSII (Fv/Fm) in all analysed plants was above the value of 0.8—equal to the optimal level determined earlier for non-stressed wild-type plants [44,45] with the highest value detected in Col-0 and Col-1 (Figure 1D, Table A1). 

Several photosynthetic parameters were measured, including photochemical quenching coefficient (qP), non-photochemical quenching (NPQ), stress-induced limitation of NPQ (qN), the effective quantum yield of PSII (Y(II)) and quantum yield of regulated energy dissipation in PSII (Y(NPQ)). The qP parameter describes the proportion of light energy absorbed by PSII to the energy used for photosynthetic phosphorylation. The highest qP values were observed in Col-0, Col-2, and Ws-2, while in the case of Col-1, the qPs’ plot curve reached its inflexion point at the lowest qP value (Figure 1D). The stress-induced limitation of NPQ parameter–qN is quite sensitive to changes in the energy status of the chloroplasts (energy-dependent quenching) and has proven to be the most sensitive parameter for the early detection of such changes. The highest value of this parameter reaching approximately 0.52 (the rate of subsequent PSII relaxation) was noted in Ler-0 and Ws-2 (Figure 1C), whereas the qN values obtained for the remaining ecotypes were similar. The analysis of the photosynthetic parameters during Light-Curve measurements showed no significant changes in the YII parameter among all accessions (Figure 1E). However, the light curves of NPQ were of higher values in Ws-2 and Col-8 amongst all accessions, which indicated higher thermal photoprotection (Figure 1F). In addition, the measurement of the Y(NPQ) parameter in both those accessions has reached the highest value (Figure 1G). Slightly increased qN levels in Ler-0 and Ws-2 and NPQ parameters in Ws-2 with simultaneous maintenance of high photosynthetic efficiency in control conditions could be the first sign of future complications in proper adjustment of the photosynthetic machinery in these two cases, amid analysed accessions, to changing light intensities [46,47].

**Figure A1:**
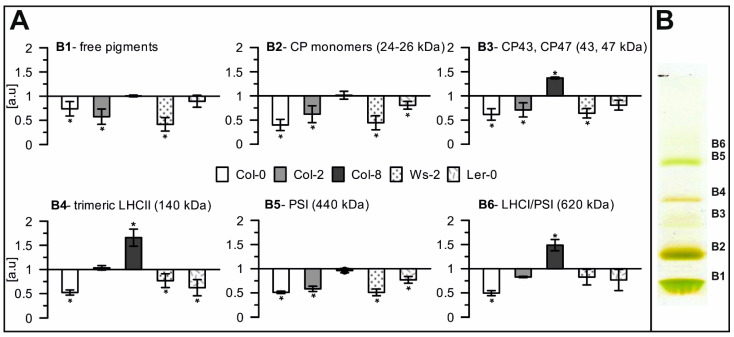
Quantitative analysis of selected photosynthetic complexes in thylakoids. (**A**)—Relative optical density of bands corresponding to particular photosynthetic complexes separated via Native-PAGE electrophoresis for accession samples in respect to Col-1, (**B**)—Visual panel for the B1–B6 symbolising specific complexes. Presented data are mean values ± SD from 3 independent experiments; pairs of results marked with an asterisk differ significantly at *p* = 0.05 (one-way ANOVA with the post hoc Tukey test).

To determine the detailed characterisation of the photosynthetic apparatus composition, Native-PAGE gel separation was used. The chlorophyll-protein complexes were gently released from the membranes by n-decyl-β-D-maltopyranoside and n-octyl-β-D-glucopyranoside. The thylakoid separation revealed six green bands assigned as follows in Figure 1B. The B6 green band is associated with undissociated LHCI–PSI complexes, while the B5 is the PSI’s reaction centre. The components of the PSII–LHCII complexes migrated as multiple green bands with increasing mobility in the gel. The B4 band corresponds to the oligomeric forms of antenna complexes, mainly to trimeric forms of LHCII; B3 band to the core antenna proteins of PSII (CP43 and CP47); and B2 to the CP monomers. Finally, a band (B1) migrated with the front and contained free pigments (Figure 1B). The bands were attributed to their components based on electrophoretic analyses in the second direction of SDS-PAGE and Western blot analysis from the team’s earlier work [14,48]. The electrophoretic patterns obtained for all Arabidopsis accessions differed in the relative intensity of bands corresponding to the LHCI–PSI complex (Figure 1B, A1; bands B6 and B5), which were detected significantly lower in the case of Ws-2 and Col-0 for the B5 and the B5 and B6 respectively. In contrast, the Col-8 accession presented a higher abundance of the LHC–PSI complex (Figure 1B, A1; band B6). The components of the PSII–LHCII (Figure 1B, A1; bands B3 and B2) were noted to decrease in comparison to Col-1, except for Col-8, which presented an increased intensity of the B3 band (Figure A1). The intensity of the region associated with LHCII trimeric complexes (Figure 1B, band B4) was detected decreased in Col-0, Ws-2, and Ler-0 and prominently increased in the Col-8 accession in respect to Col-1 (Figure A1).

The very dynamics of the photosynthesis process, measured under the established control conditions for the accessions mentioned above, is slightly different. Therefore, depending on whether one is considering, for example, research in the field of non-photochemical quenching, one can choose as the background an accession with the highest value of qN parameter, such as Ler-0 and Ws-2. On the other hand, if one wants to focus their research on analysing LHCII trimers dynamic or is interested in the components of the state-transition process (i.e., both the LHCII trimers and the PSI–LHCI photosynthetic supercomplex), it is best to choose the Col-8 accession as the genetic background for analyses.

### 2.2. The Relationship between Proteins and Lipids in Thylakoid Membranes

**Figure 2:**
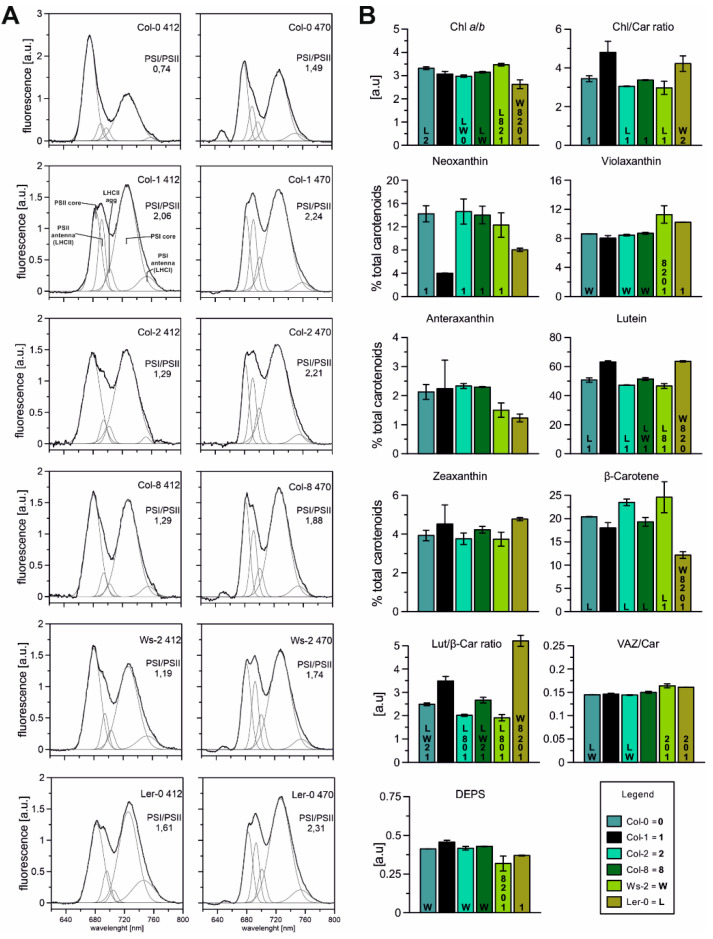
(**A**)—Spectroscopic analysis of thylakoids isolated from Arabidopsis accessions in normal conditions. Fluorescence emission spectra at 77 K, excited at 412 and 470 nm correspond to Chl*a* and Chl*b*, respectively; Chl concentration of 10 µg/mL in 20 mM HEPES-NaOH buffer (pH 7.5) containing 15 mM NaCl, 4 mM MgCl_2_, and 80% (*v*/*v*) glycerol. The spectra were normalised to the area of 100 under the spectrum. The presented spectra are representative of three separate experiments. Fluorescence emission at 685 and 695 nm corresponds to the PSII core and inner antenna, at 730 and 695 nm to the PSI core and inner antenna, at 681 to the LHCII trimers (outer PSII antennae), and 700 nm to LHCII macroaggregates. (**B**)—Composition of pigments (carotenoids and chlorophylls) extracted from isolated thylakoids in analysed Arabidopsis plants. The de-epoxidation state of the xanthophyll cycle pigments (DEPS) was calculated as follows DEPS = (Ax + Zx)/(Vx + Ax + Zx). The data are mean values ± SD from three independent experiments. Numbers or capital letters, according to legend, indicating significant differences between accessions (*p* < 0.05), i.e., Chl *a*/*b* ratio of Col-0 is significantly bigger than in Col-2 (2) and Ler-0 (L) plants.

The measurement of low temperature (77 K) fluorescence from isolated thylakoids enabled the investigation and characterisation of the photosynthetic complexes’ specifics. The typical spectrum presented in Figure 2A consists of three prominent components: the maximum at 682 nm corresponding to the emission of LHCII trimers and monomers, the PSII core visible at around 693 nm, and the PSI core at 735 nm. The Gaussian deconvolution to fluorescence spectra allowed the detection of two additional bands corresponding to aggregated LHCII and LHCI antenna forms at 702 nm and 753 nm, respectively [49]. Chl*a* is mainly bound to the chlorophyll-protein core complexes, while Chl*b* is present in peripheral antenna complexes [50]. Thus, the fluorescence emission spectra excited at 412 nm (Chl*a*) and 470 nm (Chl*b*) show changes in the relative contribution of PSI–LHCI and PSII–LHCII complexes (Figure 2A). In the spectra excited at 412 nm (Chl*a*), the calculated PSI/PSII CP complex ratio was lower than at 470 nm (Chl*b*), with the most negligible difference observed in the Col-1 accession (Figure 2A). The PSI/PSII value was estimated greater than 1 in almost all analysed ecotypes, which is related to the abundance of the PSI–LHCI complex. The exception in this instance was Col-0, where the value of the ratio at 412 nm reached 0.74 and increased slightly under the 470 nm excitation to 1.49, probably indicating a more significant proportion of the PSII–LHCII complex and lower LHCI antenna (Figure 2A; Col-0 smaller band at 753 nm). 

**Figure A2:**
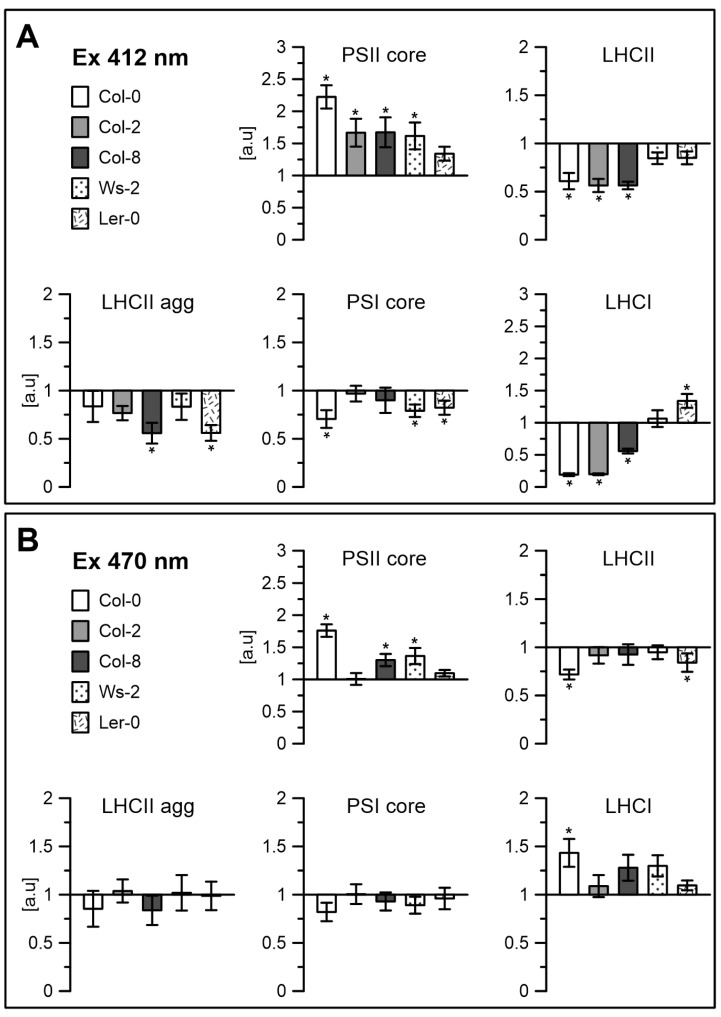
Contribution of the components: PSII core, LHCII, LHCII aggregates, PSI core and LHCI, divided using the Gaussian curve-fit, from the fluorescence emission spectra at 77 K (Figure 2A), excited at 412 nm (**A**) and 470 nm (**B**). * is *p* = 0.05.

It is surprising that despite significant differences in the protein composition of the studied accessions, the functionality of the complexes is unexpectedly similar. The detailed analysis of particular components for efficient excitation energy transfer (Figure A2) shows the Columbia population’s most significant differences. It is possible that under these conditions, in order to maintain adequate photosynthetic efficiency, the relationship between photosystems I and II is the most malleable, as it has been widely reported in studies on chlophyll *b* deficient mutants [24,51]. 

Carotenoids and chlorophylls are essential components of the thylakoid membrane responsible for stabilising and assembling CP complexes located in photosystems I and II, light-harvesting complexes, and the b_6/*f*_ complex [52]. Carotenoids are recognised to play several important physiological roles, including antenna function and photoprotection of the photosynthetic apparatus, scavenging active oxygen species, and regulating the fluidity of biomembranes [53,54]. Therefore, to estimate the physical properties of the thylakoid membranes, the pigment composition of all accessions was analysed (Figure 2B). The Chl*a*/*b* ratio was the highest in Ws-2 and the lowest in Ler-0, whereas the total Chl/Car ratio was the lowest in Col-2, Col-8, and Ws-2 (Figure 2B). Furthermore, the lowest content of neoxanthin, antheraxanthin, and β-carotene, reaching 8%, 1.23%, and 12%, respectively, was observed in Ler-0. At the same time, the zeaxanthin and lutein pools were noted the highest in this accession. The total pool of carotenoids in Col-2 and Col-8 were noted as similar. At the same time, the Ws-2 thylakoids were the most abundant in violaxanthin and β-carotene in comparison to Col-1 (Figure 2B). The lutein to the β-carotene ratio (Figure 2B; Lut/β-Car) was detected noticeably higher in Ler-0 thylakoids than Col-1. The xanthophyll cycle pigment pool (V-violaxanthin + A-anteraxanthin + Z- zeaxanthin) to the total carotenoid composition (Figure 2B; VAZ/Car) was slightly increased in the case of Ler-0 and Ws-2. DEPS levels were slightly lower in Ws-2 and Ler-0 (Figure 2B) than in Col-1.

**Figure 3:**
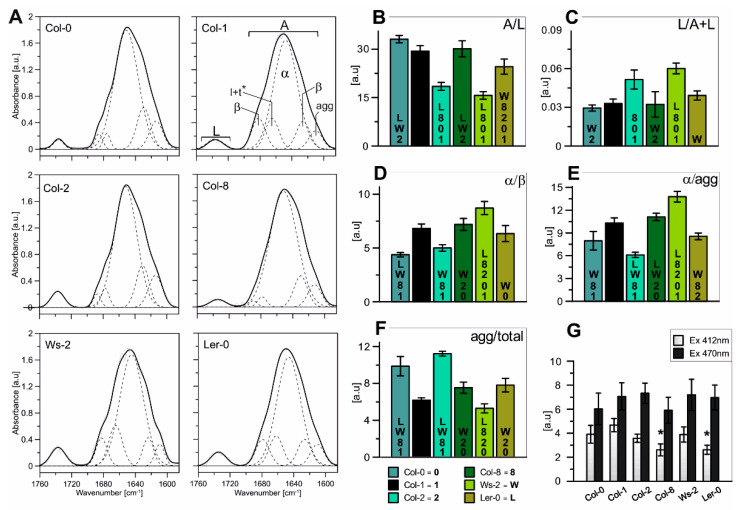
(**A**)—Normalized (to 100 AUC) FT-IR spectra in the Amide I and ester C=O regions recorded from isolated thylakoid membranes of all Arabidopsis accessions. The spectra of the Amide I region was divided into five distinct components using the Gaussian fit (shown on the Col-1 absorption spectra) and compared between analysed accessions. (**B**)—Ratios of the Amide I region to acyl lipid (ester C=O) band area (A/L). (**C**)—The relative amounts of membrane lipids (L/(A + L)). (**D**)—Ratios of the α-helis band to the parallel β-structure (α/β). (**E**)—Ratios of the α-helical band to the aggregated strands (α/agg). (**F**)—Ratio of the aggregated strands to the total band area (agg/total). (**G**)—Contribution of the LHC aggregated forms excited at 412 nm (Chl*a*) and 470 nm (Chl*b*); results marked with an asterisk differ significantly at *p* = 0.05 from the Col-1 accession. The data are mean values ± SD from three independent experiments. Numbers or capital letters, according to legend, indicating significant differences between accessions (*p* < 0.05), i.e., the A/L ratio of Col-0 is significantly bigger than in Col-2 (2), Ws-2 (W), and Ler-0 (L).

In order to determine the characteristics of the lipid–protein interactions in the thylakoid membranes, FTIR spectroscopy measurements were made (Figure 3). The spectrum range between 1700 and 1580 cm^−1^ is related to the Amide I region (Figure 3A; marked with A symbol) and corresponds to the vibration of the peptide bond carbonyl group, while the band localised between 1760 and 1710 cm^−1^ is correlated with the acyl lipid (ester C=O) vibrations (Figure 3A; marked with L symbol). Therefore, the Amid I to ester C=O band intensity ratio (Figure 3B; A/L) is a valuable measure of changes in the relative protein to lipid ratio [55]. Our results revealed that the A/L was similar in Col-0, Col-1, and Col-8, whereas the ratio value was lower in Ler-0, Ws-2, and Col-2. Furthermore, obtained results were consistent with the analysis of the relative amounts of membrane lipids (Figure 3C; L/(A + L)), suggesting an increased level of the lipid component in Ws-2 and Col-2 thylakoids in comparison to Col-1. 

Furthermore, the spectra region characteristic for the proteins secondary structure (Figure 3A; A-Amide I region) was deconvoluted into several distinctive components using the Gaussian curve-fit and marked on Figure 3A. The main band centred at 1650 cm^−1^ is associated with an α-helical protein structure and is accompanied by bands located around 1690 cm^−1^ and 1675 cm^−1^ corresponding to antiparallel β-structures and turns and loops, respectively [9,56]. Additionally, the spectral region around 1610 cm^−1^ is ascribed to aggregated strands, while the 1621 cm^−1^ band is associated with the formation of hydrogen bonds between α-helices of neighbouring proteins formally assigned to the parallel β-structure [56,57]. The generated ratios of the α-helical band to the parallel β-structure (Figure 3D) and the aggregated strands (Figure 3E) reflect the level of internal and external thylakoid protein aggregation [56]. The tendency of the changes of those two parameters was detected similar among accessions: noticeably lower in the case of Col-0 and Col-2 and increased in Ws-2 in comparison to Col-1 (Figure 3D,E). The Col-2 thylakoids presented the lowest level of α/agg ratio (Figure 3E). The agg/total ratio presented the contribution of the aggregated strands to the total band area and was most prominent in the case of Col-0 and Col-2 (Figure 3F), suggesting higher interactions between proteins in the thylakoid membranes. While the values of LHC aggregated forms particularly excited at 412 nm (Chl*a*) were detected noticeably lower in Col-8 and Ler-0 thylakoids (Figure 3G). 

The higher content of the lipid membrane component of the Col-2 and Ws-2 thylakoids may indicate greater overall fluidity of these membranes or more significant regions with fewer proteins present. Hence, faster/better migration of the moving parts of the photosynthetic puzzle and faster response to changing conditions.

### 2.3. PSI and PSII Gene Expression

**Figure 4:**
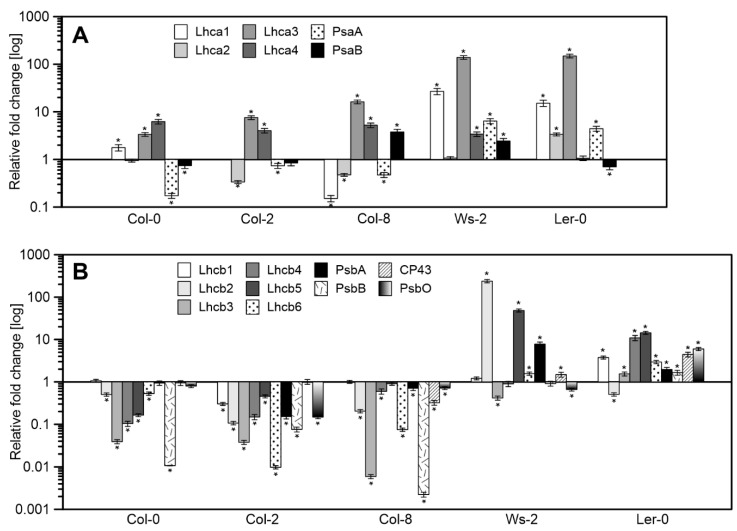
Relative mRNA levels of genes corresponding to antenna and core proteins of PSI (**A**) and PSII (**B**) in reference to the Col-1 as arbitrarily chosen control. The data are mean values ± SD from three to four independent experiments; results marked with an asterisk differ significantly at *p* = 0.05 from the Col-1 accession.

The transcript accumulation of genes corresponding to antenna and core proteins of PSII and PSI were measured (Figure 4) in reference to the Col-1 accession. The mRNA levels of all the PSI antenna genes were significantly increased in Ws-2 and Ler-0, while the Columbia ecotypes presented increased levels of Lhca3 and Lhca4 (Figure 4A). Conversely, the PsaA core subunit mRNA levels decreased in Col-0, Col-2, and Col-8 compared to Ws-2 and Ler-0 (Figure 4A).

The expression levels of genes corresponding to PSII antenna proteins were severely decreased in Columbia accessions (Figure 4B) in relation to Col-1. While in Ws-2, significantly increased mRNA levels of Lhcb2, Lhcb5, and PsbA were noted. Ler-0 presented an increase in the expression values correlated with the PSII core and antenna proteins except for Lhcb2 (Figure 4B). The Columbia accessions showed a similar gene expression pattern as in Ler-0 and Ws-2.

Most of the genes encoding the antennas of photosystem II are elevated in Ler-0 and Ws-2. Moreover, the same plants that responded to light with high qN levels in the early stages of illumination have significantly lower DEPS than the measured Col-n. Therefore, they can be an interesting object in research on the rearrangement of antenna systems, post-translational modification processes of antenna proteins, or antenna–xanthophyll interactions.

## 3. Discussion

The wide geographical distribution of *A. thaliana* accessions is reflected in their remarkable adaptability and a vast diversity of forms. It is believed that ecotype plasticity could be a result of a unique arrangement of their photosynthetic apparatus. Thus, in this paper, various functional, spectral, biophysical, and transcriptomic methods were employed to characterise and compare the photosynthetic abilities of six Arabidopsis accessions in control growth conditions.

### 3.1. Diversity in the Thylakoid Membrane CP Complex Distribution Amongst Accessions

Low-temperature fluorescence results (Figure 2A) and Native-PAGE analysis of gels (Figure 1B) revealed differences in the relative amounts of the photosynthetic complexes in the thylakoids isolated from all analysed accessions. In Col-0 and Ws-2, a lower intensity of the band corresponding to the PSI–LHCI complex was detected (Figure 1B; band B6). The lower value of PSI/PSII ratio noted at the 77 K emission spectra in both accessions (Figure 2A) was in line with those results, suggesting a distinct distribution of the PSI–LHCI complex compared to the other analysed plants. The FTIR results obtained for the Col-0 thylakoids confirmed this statement, as the band equivalent to the parallel β structure in the Amide I region was detected as being the highest (Figure 3) and reflected changes in the lamellar protein interactions. While, the increased Chl*a*/*b* ratio registered in the Ws-2 thylakoids and reduced Chl/Car ratio (Figure 2B), also stated before [58], could be associated with ultrastructural changes of thylakoid membranes in this accession. Thus, Yin et al., 2012 has reported reduced grana height and width in the Ws compared to Col-0 and Ler-0 [58]. Interestingly, all three accessions demonstrated depleted amounts of the LHCII trimers, visible both on Native-Page gels (Figure 1B, band B4 and Figure A1) and 77 K fluorescence spectra (Figure 2A; band at 682 nm). The abundance of LHC protein in thylakoids is strictly associated with grana size, as it has been shown in the case of *ch1* mutants lacking most of the Lhcb proteins to result in severe grana decrease [59]. 

### 3.2. Different Patterns of mRNA Transcription Levels

We further investigated whether the altered proportions detected in chlorophyll-protein complexes were associated with the expression level of genes corresponding to PSI and PSII proteins (Figure 4). Coordination of the photosynthetic gene expression requires the transcription of genetic information located in the nucleus and plastid genome, for which multiple regulation pathways exist [60]. Still, our understanding of the integration between photosynthetic gene transcription and protein accumulation is volatile [61,62,63]. So far, evidence of such connections is being linked with short-term acclimatory responses to changing light intensities [29,64,65]. Meanwhile, the post-transcriptional rather than transcriptional regulation of genes involved in photosynthesis, especially LHC genes, is believed to control the protein levels during long-term acclimation [63,66].

Nevertheless, the presented results indicate differences among accessions involved with the assembly of the photosynthetic machinery already at the transcriptional level, probably due to their genetic variation features. Correlation in the transcriptional regulation pattern between Ws-2 and Ler-0 in contrast to the Columbia line accessions (Col-0, Col-2, and Col-8) was registered. However, the mRNA levels did not fully correspond to the proportion of CP complexes received via the 77 K fluorescence results, implying an action of other regulatory mechanisms at the post-transcriptional level.

### 3.3. Links between Lipid–Protein Interactions, Carotenoid Composition, and NPQ Capacity

FTIR spectroscopic analysis revealed higher lipid-to-protein proportion in the thylakoid membranes of Col-2 and Ws-2 (Figure 3B,C). It was consistent with the significantly lower Chl/Car ratio detected in these accessions (Figure 2B). The thylakoid membrane fluidity can be determined by various factors [9], which involves the content and orientation of carotenoids located in the thylakoid lipid phase [67]. The substantially lower value of the thylakoid membrane rigidification parameter, Lut/β-Car [53,68] detected in Ws-2 and Col-2 (Figure 2B), combined with increased lipid-to-protein ratio, indicated increased thylakoid membrane mobility in those accessions that could lead to the less organised arrangement of CP complexes. This conclusion suggests the more effortless lateral movement of the PSII repair cycle proteins in harmful light intensities and could explain the divergent distribution of the PSI–LHCI in Ws-2 mentioned previously. In contrast, Ler-0 thylakoids were found to be more rigid, in line with previous findings presented by Yin et al., 2012 [58]. At the same time, results obtained by Flood et al., 2014 [42] presented lower D1 and LHCII phosphorylation rates in the Ler accession compared to Ws associated with diverse lateral mobility in the membrane.

Despite variations in the Lut/β-Car proportion, Ler-0 and Ws-2 displayed comparable amounts of xanthophyll cycle pigments with an increased VAZ/Car ratio than other accessions (Figure 2B). Since we stated that both accessions presented lower LHCII amounts, the xanthophyll distribution must have been divided less extensively to LHCII and more towards LHCI antennas with a small pool present in the lipid matrix of the thylakoid membranes [12]. Therefore, the additional violaxanthin pool in the Ler-0 accession was probably involved with the LHCI antennas in the PSI–LHCI complex due to a higher detected PSI/PSII ratio. What is more, inferring from the obtained data, the non-bound violaxanthin pool could take part, next to lutein, in stabilising the thylakoid bilayer by creating a more rigid environment, as proposed for amphiphilic xanthophylls [13,68]. Increased qN values reported for Ler-0 (Figure 1C) in control conditions might indicate limitations for this accession in adjusting the photosynthetic apparatus to changing light intensities. Higher qN, NPQ, and Y(NPQ) parameters observed in Ws-2 accession indicate cooperation with xanthophyll cycle pigments and activation of photoprotective mechanisms [12,46]. However, low DEPS value and no difference in the effective quantum yield of PSII (Figure 1E) parameter suggest that the violaxanthin pool in this accession could enhance the PSII quantum efficiency by additionally bounding to the LHCII antenna as it was observed for the *lut2npq1* mutant [69]. Taken together, the thylakoid membrane lipid-to-protein proportion, and the Chl/Car and VAZ/Car ratios seemed to determine the changes in photosynthetic efficiency and the CP complexes’ distribution observed amongst accessions with the most prominent variations noticed in the Ws-2 and Ler-0.

## 4. Materials and Methods

### 4.1. Plant Materials and Growth Conditions

*Arabidopsis thaliana* plants (ecotype Columbia: Col-0, Col-1, Col-2, Col-8, *Landsberg erecta*—Ler-0, and Wasilevskaja—Ws-2) were grown in hydroponic culture using seed holders (system from Araponics SA, Liege, Belgium) in custom 1.8-L boxes with low-density support. The seeds were placed on seed holders containing 0.65% (*w*/*v*) agar (Sigma Aldrich Inc., Saint Louis, MO, USA) plugs. The boxes were filled with suitably diluted General Hydroponics solution (GH Flora Series, Hawthorne, Vancouver, WA, USA). Plants were grown for 8–10 weeks in an 8-h photoperiod at 22 °C/18 °C (day/night) at PAR 110 µmol photons m^–2^ s^–1^.

### 4.2. Photosynthetic Measurements. Analysis by Pulse-Amplitude Modulated Fluorescence of Chlorophyll a

In vivo chlorophyll *a* fluorescence was measured using a PAM-2000 chlorophyll fluorometer (Heinz Walz, Effeltrich, Germany). Plants were dark-adapted for 30 min. First, the minimum chlorophyll fluorescence at open PSII centres in the dark (Fo) and under actinic light (Fo’) was determined using a weak, red measuring light (650 nm) with very low intensity (0.8 μmol m^−2^ s^−1^). Next, a saturating pulse of white light (3000 μmol m^−2^ s^−1^ for 800 ms) was applied to estimate the maximum chlorophyll fluorescence at closed PSII centres in the dark (Fm) and under actinic light (Fm’). The parameters of Fv/Fm, qP, qN, YII, NPQ, and Y(NPQ) were calculated as described in [44]. The build in the program for determination of induction curve and subsequently measured light curve (IC + LC) was used on whole leaves. 

### 4.3. Preparation of Thylakoid Membranes

As described previously, thylakoid membranes were isolated by homogenising leaves in a buffered isotonic medium [48]. Thylakoid membranes were always freshly prepared before each experiment and were kept on ice and in the dark for subsequent use. Chlorophyll concentration (Chl) was quantified spectrophotometrically after extraction with 80% acetone (Merck KGaA, Darmstadt, Germany) according to the method of [70].

### 4.4. Fourier-Transform Infrared Spectroscopy (FTIR) Measurements

Thylakoid membranes isolated from *A. thaliana* accessions were resuspended in a D_2_O-based 20 mM Hepes–NaOH (pH 7.0) buffer containing 330 mM sorbitol, 15 mM NaCl, and 4 mM MgCl_2 (_Merck KGaA, Darmstadt, Germany) and then centrifuged at 7200× *g* for 10 min at 4 °C (Beckman Coulter, Brea, CA, USA). This step was repeated three times to replace the H_2_O-based buffer with a D_2_O one (Sigma Aldrich Inc., Saint Louis, MO, USA). Fourier-transform infrared (FTIR) spectra were recorded on a Nicolet FTIR Spectrometer (Thermo Scientific, Waltham, MA, USA). Sample measurements and data analysis were performed as described previously [56].

### 4.5. Low Temperature (77 K) Fluorescence Measurements

Low temperature (77 K) fluorescence emission spectra were recorded using a modified Shimadzu RF-5301PC spectrofluorimeter (Shimadzu Corp., Kioto, Japan), where optical fibres led to excitation and emission beams. Thylakoids were placed in a metal cuvette and submerged in liquid nitrogen. The excitation wavelength was set at 412, and 470 nm, excitation and emission slits at 5 nm and scans were taken in the range of 600 to 800 nm. Each spectrum was recorded twice, averaged, background corrected, and the obtained curve was shifted to 0 at points 640 and 780 nm.

### 4.6. Mild-Denaturing Electrophoresis

Isolated thylakoid membranes were prepared and separated via mild-denaturing electrophoresis as described previously [71], with slight modification—samples containing 8.3 µg of chlorophyll were loaded into each well of the stacking gel [72].

### 4.7. Pigment Extraction and Separation

Pigments were extracted as described previously [73]. The HPLC analysis of pigments was carried out by the method from Sztatelman et al., 2015 [74]. An amount of 30 µL of methanol pigment extract was loaded with a loop onto a C-18 column (Bionacom Velocity, 5 microns, 4.6 × 250 mm, BIONACOM LTD, Coventry, UK). Pigments were identified by retention time compared to standards. The chromatogram analysis and peak retention were conducted using MassLynx software (v.4.1, Waters Corp., Milford, MA, USA). The de-epoxidation state of the xanthophyll cycle pigments (DEPS) was calculated as follows DEPS = (Ax + Zx)/(Vx + Ax + Zx) according to [75].

### 4.8. PCR Analysis

Total RNA extraction, selection of the optimal reference genes, and expression analysis via quantitative PCR in a MyGo Pro Real-Time PCR thermocycler (IT-IS INTERNATIONAL LTD., Stokesley, UK) were conducted as described in Wójtowicz et al., 2020 [72]. Primer sets were taken from Xu and coworkers [76] or designed using Primer-BLAST (NCBI, Bethesda, MD, USA) and checked for specificity by BLAST searching the *A. thaliana* RefSeq RNA database [72].

## 5. Conclusions

In this study, we examined the photosynthetic abilities and performance of six *Arabidopsis thaliana* accessions grown in control conditions to distinguish characteristic disparities accountable for their remarkable plasticity to adapt in various conditions. Using a functional and physiological approach, we have established that despite no visible differences in the photosynthetic efficiency, the machinery behind it reveals its unique composition. The obtained data showed that among the group of analysed accessions, Ler-0 and Ws-2 displayed noticeable changes in the thylakoid membrane composition, yet no difference in photosynthetic efficiency. Furthermore, the opposite values of the lipid-to-protein contribution, Chlorophyll-to-Carotenoid ratio (Chl/Car), and distinct xanthophyll cycle pigment distribution had determined the changes in the arrangement of the CP complexes, PSI/PSII ratio, and lateral mobility of the thylakoid membrane. In addition, a correlation in the transcript level of genes corresponding to PSI and PSII proteins in these two accessions was apparent. However, the importance of this information requires further elucidation. 

Our results provide elementary yet valuable information about the characteristics of the changes detected in the photosynthetic apparatus of analysed accessions and the scale of these disparities, proving that selecting a suitable background line in future experiments requires literature verification. Considering all the data differences discussed in this paper, a genetic blueprint for photosynthesis in lipid/protein thylakoid ratio and carotenoid content exists within *A. thaliana* accessions.

## Data Availability

Data available on request due to restrictions eg privacy. The data presented in this study are available on request from the corresponding author.

## Appendix A

**Table A1:** The efficiency of PSII–Fv/Fm. Data are mean values ± SD from at least three independent experiments.

	Col-0	Col-1	Col-2	Col-8	Ws-2	Ler-0
Fv/Fm	0.827	0.824	0.819	0.821	0.814	0.815
	±0.005	±0.007	±0.008	±0.005	±0.013	±0.007
